# Supplementary material for: Free-Radical Photopolymerization of Acrylonitrile Grafted onto Epoxidized Natural Rubber
Source: Polymers (Basel). 2021 Feb 23;13(4):660. doi: 10.3390/polym13040660 (PMC7926637; doi:10.3390/polym13040660)
Supplement: Supplementary file 1 [file polymers-13-00660-s001.pdf]

polymers

MDPI

Supplementary Materials

Free-Radical Photopolymerization of Acrylonitrile Grafted onto Epoxidized Natural Rubber

Rawdah Whba [1,2], Mohd Sukor Su'ait [3], Lee Tian Khoon [1,*], Salmiah Ibrahim [4], Nor Sabirin Mohamed [4] and Azizan Ahmad [1,5,*]

1 Department of Chemical Sciences, Faculty of Sciences and Technology, Universiti Kebangsaan Malaysia, 43600 Bangi, Selangor, Malaysia; rawdahukm31@gmail.com
2 Department of Chemistry, Faculty of Applied Sciences, Taiz University, Taiz, Yemen
3 Solar Energy Research Institute (SERI), Universiti Kebangsaan Malaysia, 43600 Bangi, Selangor, Malaysia; mohdsukor@ukm.edu.my
4 Centre for Foundation Studies in Science, University of Malaya, 50603 Kuala Lumpur, Malaysia; nsabirin@um.edu.my (N.S.M.); salmiah01@um.edu.my (S.I.)
5 Research Center for Quantum Engineering Design, Faculty of Science and Technology, Universitas Airlangga, Surabaya, Indonesia

* Correspondence: tiankhoon@ukm.edu.my (L.T.K.); Tel.: +60 12-7279286 (L.T.K.); azizan@ukm.edu.my (A.A.); Tel.: +6019-3666576 (A.A.)

Citation: Whba, R.; Su'ait, M.S.; Tian Khoon, L.; Ibrahim, S.; Mohamed, N.S.; Ahmad, A. Free-Radical Photopolymerization of Acrylonitrile Grafted onto Epoxidized Natural Rubber. *Polymers* **2021**, *13*, 660. https://doi.org/10.3390/polym13040660

Academic Editor: Vicente Compañ Moreno

Received: 15 January 2021

Accepted: 16 February 2021

Published: 23 February 2021

Publisher's Note: MDPI stays neutral with regard to jurisdictional claims in published maps and institutional affiliations.

Copyright: © 2021 by the authors. Licensee MDPI, Basel, Switzerland. This article is an open access article

distributed under the terms and conditions of the Creative Commons Attribution (CC BY) license (http://creativecommons.org/licenses/by/4.0/).

Table S1. FTIR's wavenumber assignments of ENR- 25, ACN, PAN, and ACN-g-ENR products at different mole ratios.

Functional Group	Wavenumber (cm^{-1})					
	ENR 25	ACN	PAN	ACN_{10}-g-ENR_1	ACN_{15}-g-ENR_1	ACN_{20}-g-ENR_1
C-H stretch	Nd	3070	Nd	Nd	Nd	Nd
Sym. stretching (υ_s -CH_3)	2962	Nd	Nd	2962	2962	2962
Asym. stretching (υ_{as} -CH_2)	2924	2918	2916	2924	2924	2924
Sym. stretching (υ_s -CH_2)	2855	2852	2845	2855	2855	2855
in-plane bending (scissoring) (δs-CH_2)	1449	1418	1455	1447	1447	1447
out-of-plane bending (wagging) (ω -CH_2)	1379	Nd	1366	1375	1375	1375
Epoxy, whole ring stretching	1250	Nd	Nd	1250	1250	1250
in-plane bending (rocking) (ρ -CH_2)	738	Nd	Nd	Nd	751	753
=C-H out - plane deformation vib.	835	Nd	802	834	832	832
-CH wagging	Nd	684	Nd	659	659	659
-C≡ N stretch	Nd	2230	2242	2241	2242	2241
-C=N stretch	Nd	1713	1728	1710	1717	1723
-C=C stretch	1664	1602	1655	1670	1670	1678
Epoxy, half ring stretching	870	Nd	Nd	870	870	870

Nd: Not detected.

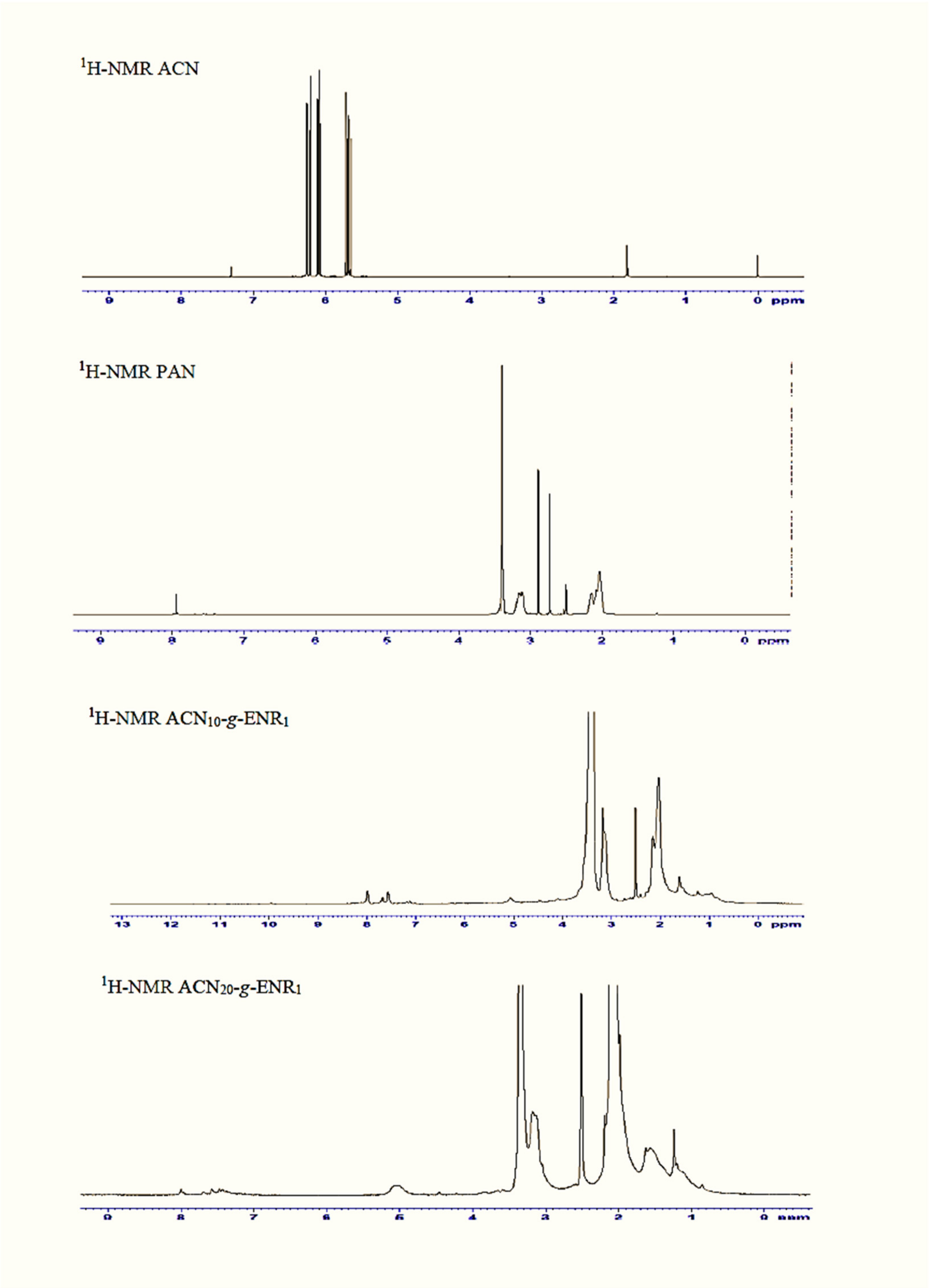

Figure S1. 1D ^{1}H-NMR spectra for ACN, PAN, ACN_{10}-*g*-ENR_1, and ACN_{20}-*g*-ENR_1.

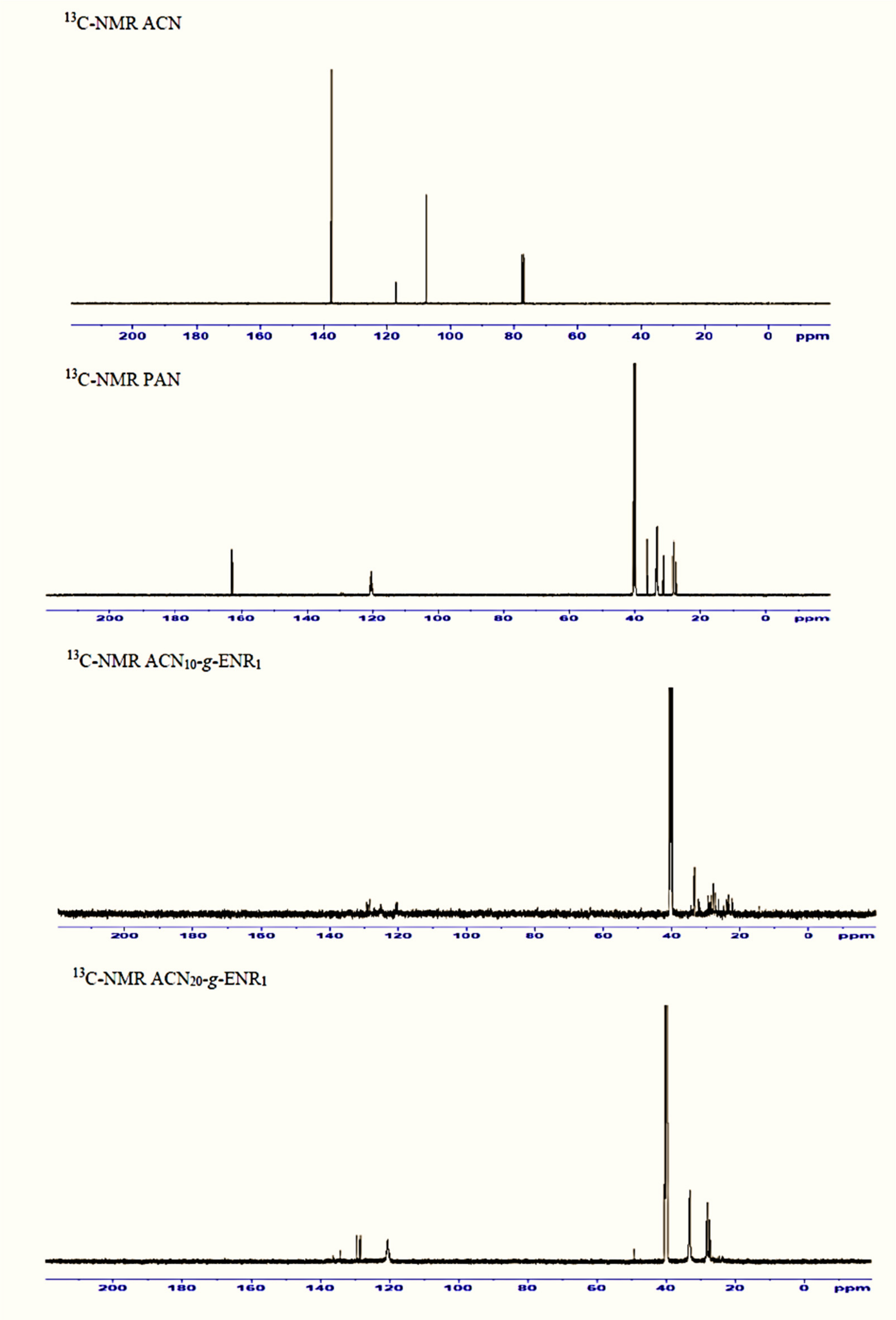

Figure S2. 1D ^{13}C-NMR spectra for ACN, PAN, ACN_{10}-*g*-ENR_1, and ACN_{20}-*g*-ENR_1.

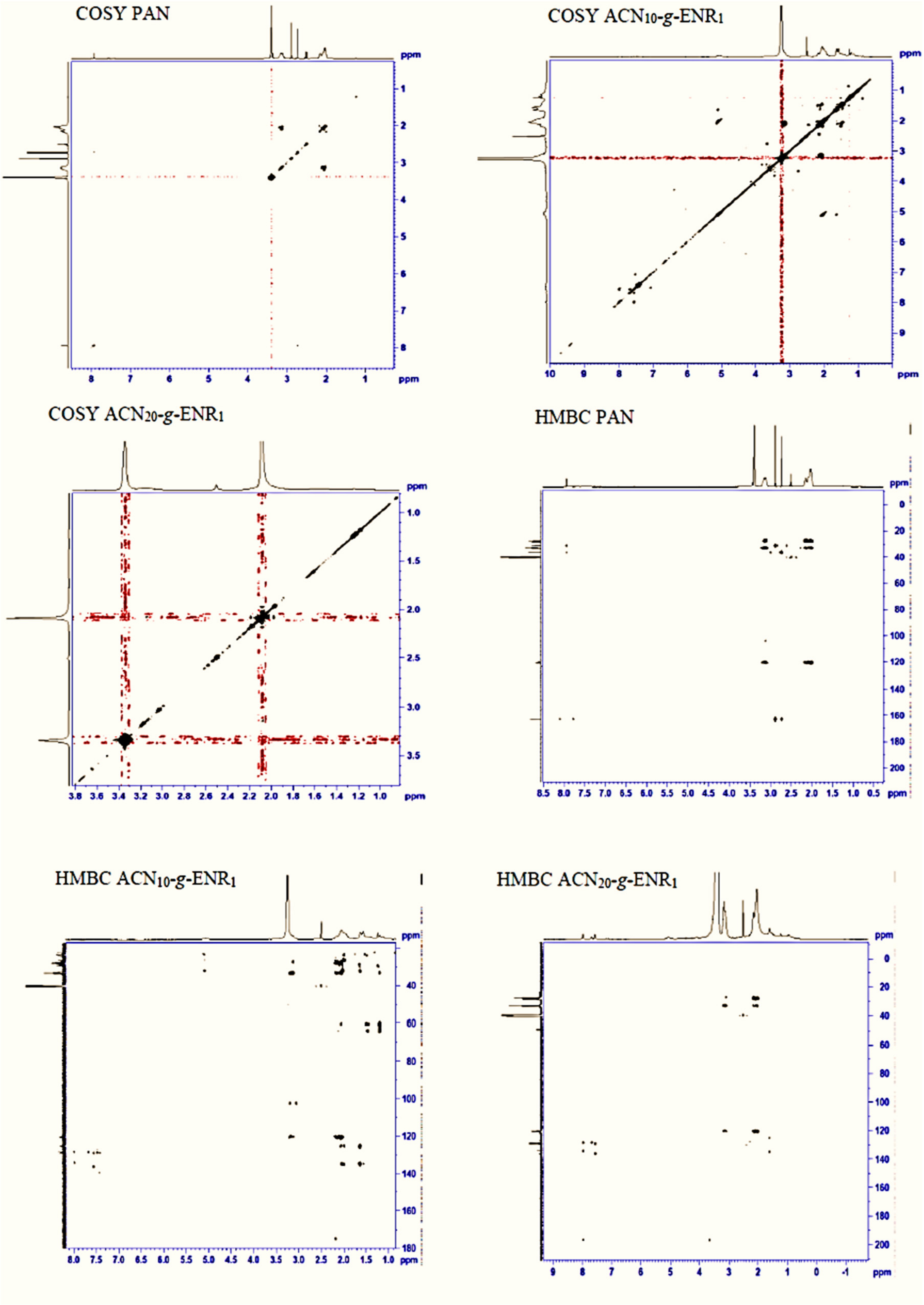

Figure S3. 2D NMR spectra for ACN, PAN, ACN_{10}-*g*-ENR_1, and ACN_{20}-*g*-ENR_1.

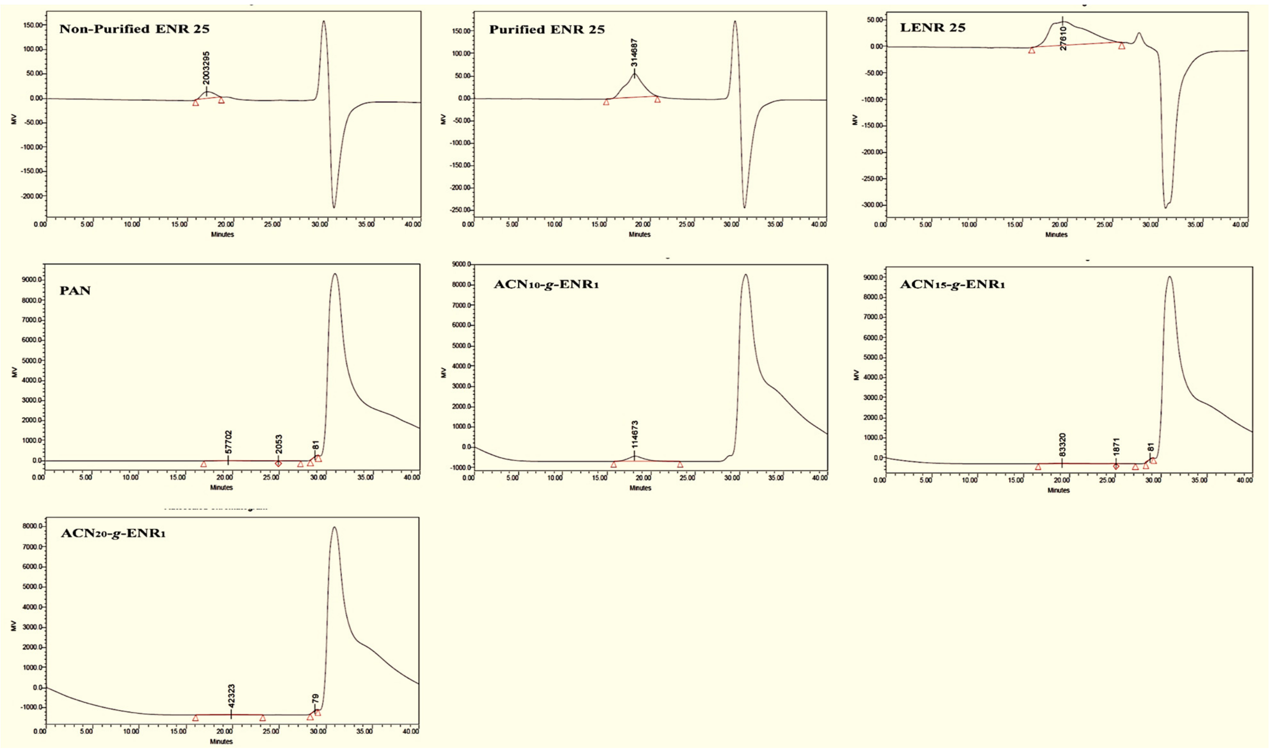

Figure S4. Autoscaled Chromatogram GPC of PAN, LENR 25, ENR- 25, ACN_{10}-*g*-ENR_1, ACN_{15}-*g*-ENR_1, and ACN_{20}-*g*-ENR_1.

Table S2. % GY and % GE data of PAN, ENR- 25 and ACN-*g*-ENR products at various mole ratios using Soxhlet method.

Sample ID	% GY	% GE
PAN	31.70	3.71
ENR- 25	N/A	17.63
ACN_{10}-*g*-ENR_1	32.18	21.08
ACN_{15}-*g*-ENR_1	64.05	56.59
ACN_{20}-*g*-ENR_1	49.78	16.98

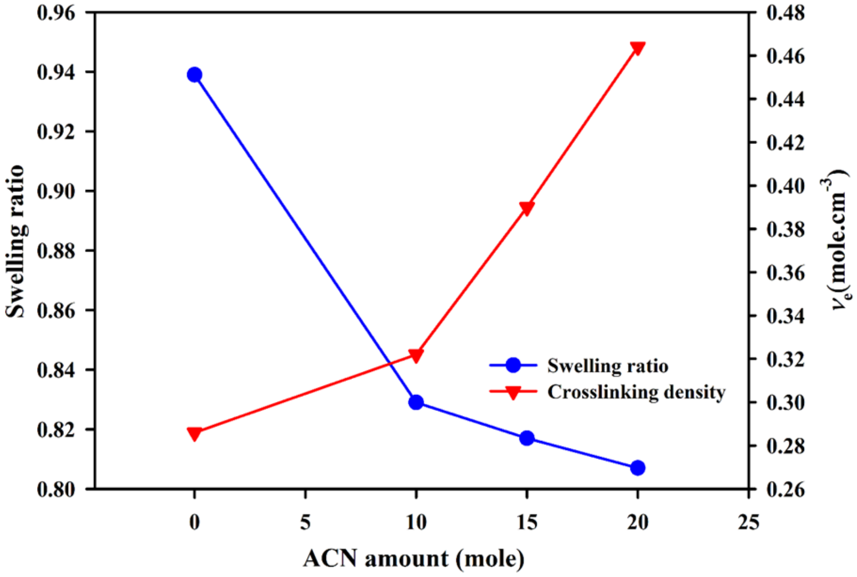

Figure S5. Swelling ratio and crosslink density of ACN-*g*-ENR products at various mole ratios using the equilibrium swelling method.

Table S3. A summary of thermo-mechanical properties as well as the dielectric properties for NR, ENR- 25, ENR- 50, and NBR from the previous studies compared to ACN-*g*-ENR products with various mole ratios.

Parameter	Polymers						
	NR	ENR- 25	ENR- 50	NBR	ACN_{10}-*g*-ENR_1	ACN_{15}-*g*-ENR_1	ACN_{20}-*g*-ENR_1
M_n (g mole $^{-1}$)	262,000 [1]	178,792	263,029	100,600 [2]	50,408	11,286	33,547
M_w (g mole $^{-1}$)	830,000 [1]	1,287,538	1,376,572	40,000 – 1,550,00 [3]	171,064	114,657	252,786
PDI (M_w/ M_n)	3.2 [1]	7.20	5.23	NA	3.39	10.16	7.54
E (MPa) at -60 °C	6700 [4]	6200 [4]	3800 [4]	~ 100 [5]	22,286	19,779	132,353
E (MPa) at 25 °C	1.6 [4]	2.1 [4]	1.9 [4]	~ 1 [5]	713	55	56,222
Tan δ_{max} (DMA)	2.48 [4]	2.62 [4]	2.70 [4]	~ 1.3 [5]	0.37	1.05	0.34
T_g °C (DMA)	-49 [4]	-24 [4]	-5.7 [4]	-19 [5]	-12	-15.73	13
T_g °C (DSC)	-67 [6]	-44	-21 [6]	-21.7	-34.96	-39.25	-34.39
T_d (°C)	300 [7]	321	388 [8]	340 [2]	333	393	368
T_{max} (°C)	~ 395 [7]	397	393 [8]	520 [2]	400	409	411
Conductivity (σ, S cm^{-1})	10^{-13} [9]	10^{-11} [10]	10^{-11} [11]	6.2×10^{-11} [12]	1.02×10^{-10}	4.45×10^{-9}	3.43×10^{-9}
ε_r	2.22 [4]	3.76 [4]	6.00 [4]	~ 10.8 [13]	2.05	8.04	6.71
ε_i	0.01 [4]	0.03 [4]	0.25 [4]	~ 0.03 [13]	0.016	10.40	6.87
Tan δ	0.006 [4]	0.008 [4]	0.042 [4]	~ 0.044 [13]	0.008	1.294	1.023

* The dielectric behavior was recorded at 100 Hz while NR, ENR 25, ENR 50, and our work have measured at 1 kHz.

References

1. Utara, S.; Boochathum, P. Effect of molecular weight of natural rubber on the compatibility and crystallization behavior of LLDPE/NR blends. *Polym. Plast. Technol. Eng*. **2011**, *50*, 1019–1026.
2. Samantarai, S.; Mahata, D.; Nag, A.; Nando, G.B.; Das, N.C. Functionalization of acrylonitrile butadiene rubber with meta-pentadecenyl phenol, a multifunctional additive and a renewable resource. *Rubb, Chem. Technol*. **2017**, *90*, 683–698.
3. Dürr, C.J.; Hlalele, L.; Schneider-Baumann, M.; Kaiser, A.; Brandau, S.; Barner-Kowollik, C. Determining the Mark-Houwink parameters of nitrile rubber: A chromatographic investigation of the NBR microstructure. *Polym. Chem*. **2013**, *4*, 4755–4767.
4. Salaeh, S. Processing of Natural Rubber Composites and Blends: Relation between Structure and Properties. Ph.D. Thesis, Université Claude Bernard Lyon 1, Villeurbanne, France; Prince of Songkla University, Pattani Thailand, 4 July 2014.
5. Ramesan, M.T.; Alex, R. Compatibilization of SBR/NBR blends using chemically modified styrene-*co*-butadiene rubber Part 2. Effect of compatibilizer loading. *Polym. Int*. **2001**, *50*, 1298–1308.
6. Harun, F.; Chan, C.H. *Electronic Applications of Polymer Electrolytes of Epoxidized Natural Rubber and Its Composites*; Springer: Cham, Switzerland, 2016; pp. 37–59.
7. Silva, M.J.D.; Sanches, A.O.; Malmonge, L.F.; Malmonge, J.A. Electrical, mechanical, and thermal analysis of natural rubber/polyaniline-Dbsa composite. *Mater. Res*. **2014**, *17*, 59–63.
8. Rosniza, H.; Bakar, M.A.; Hamid, S.A.; Ismail, J. Cyclopentyl trisilanol silsesquioxanes-modified natural rubber (CpSSQ $(OH)_3$–ENR-50) nanocomposite in the presence of tin (II) chloride dihydrate. *Indones. J. Chem*. **2007**, *7*,111–116.

9. Aguilar-Bolados, H.; Yazdani-Pedram, M.; Brasero, J.; Lopez-Manchado, M.A. Influence of the surfactant nature on the occurrence of self-assembly between rubber particles and thermally reduced graphite oxide during the preparation of natural rubber nanocomposites. *J. Nanomater.* **2015**, *2015*, doi:10.1155/2015/212493.
10. Hussin, N.S.; Harun, F.; Chan, C.H. Thermal Properties and Conductivity of Thermally Treated Epoxidized Natural Rubber-Based Solid Polymer Electrolytes. *Macromol. Symp.* **2017**, *376*, 1700049.
11. Zainal, N.; Mohamed, N.S.; Idris, R. Properties of ENR-50 based electrolyte system. *Sains Malays.* **2013**, *42*, 481–485.
12. Yang, D.; Ruan, M., Huang, S.; Wu, Y.; Li, S.; Wang, H.; Shang, Y.; Li, B.; Guo, W.; Zhang, L. Improved electromechanical properties of NBR dielectric composites by poly(dopamine) and silane surface functionalized TiO_2 nanoparticles. *J. Mater. Chem. C* **2016**, *4*, 7724–7734.
13. Yang, D.; Kong, X.; Ni, Y.; Gao, D.; Yang, B.; Zhu, Y.; Zhang, L. Novel nitrile-butadiene rubber composites with enhanced thermal conductivity and high dielectric constant. *Compos. Part A Appl. Sci. Manuf.* **2019**, *124*, 10544.
